# Predictors of adherence to exercise interventions in people with schizophrenia

**DOI:** 10.1007/s00406-024-01789-w

**Published:** 2024-03-29

**Authors:** Rebecca Schwaiger, Isabel Maurus, Moritz Lembeck, Irina Papazova, David Greska, Susanne Muenz, Eliska Sykorova, Cristina E. Thieme, Bob O. Vogel, Sebastian Mohnke, Charlotte Huppertz, Astrid Roeh, Katriona Keller-Varady, Berend Malchow, Henrik Walter, Bernd Wolfarth, Wolfgang Wölwer, Karsten Henkel, Dusan Hirjak, Andrea Schmitt, Alkomiet Hasan, Andreas Meyer-Lindenberg, Peter Falkai, Lukas Roell

**Affiliations:** 1 Department of Psychiatry and Psychotherapy, LMU University Hospital, LMU Munich, Nussbaumstrasse 7, 80336 Munich, Germany; 2 Department of Psychiatry, Psychotherapy and Psychosomatics of the University Augsburg, Medical Faculty, University of Augsburg, Bezirkskrankenhaus Augsburg, Augsburg, Germany; 3 Central Institute of Mental Health, Medical Faculty Mannheim, Heidelberg University, Heidelberg, Germany; 4 Department of Psychiatry and Psychotherapy, University Hospital Charité Berlin, Berlin, Germany; 5 Department of Sports Medicine, University Hospital Charité Berlin, Berlin, Germany; 6 Department of Psychiatry, Psychotherapy and Psychosomatics, RWTH Aachen University, Aachen, Germany; 7 Clinic of Rehabilitation and Sports Medicine, Hannover Medical School, Hannover, Germany; 8 Department of Psychiatry and Psychotherapy, University Hospital Göttingen, Göttingen, Germany; 9 Department of Psychiatry and Psychotherapy, Medical Faculty, Heinrich-Heine University, Düsseldorf, Germany; 10 Laboratory of Neuroscience (LIM27), Institute of Psychiatry, University of Sao Paulo, São Paulo, Brazil; 11 Max Planck Institute of Psychiatry, Munich, Germany; 12 DZPG (German Center for Mental Health), Partner Site Munich/Augsburg, Munich/Augsburg, Germany

**Keywords:** Schizophrenia, Exercise, Adherence, Randomized-controlled trial, Machine learning

## Abstract

Exercise interventions are nowadays considered as effective add-on treatments in people with schizophrenia but are usually associated with high dropout rates. Therefore, the present study investigated potential predictors of adherence from a large multicenter study, encompassing two types of exercise training, conducted over a 6-month period with individuals with schizophrenia. First, we examined the role of multiple participants’ characteristics, including levels of functioning, symptom severity, cognitive performance, quality of life, and physical fitness. Second, we used *K*-means clustering to identify clinical subgroups of participants that potentially exhibited superior adherence. Last, we explored if adherence could be predicted on the individual level using Random Forest, Logistic Regression, and Ridge Regression. We found that individuals with higher levels of functioning at baseline were more likely to adhere to the exercise interventions, while other factors such as symptom severity, cognitive performance, quality of life or physical fitness seemed to be less influential. Accordingly, the high-functioning group with low symptoms exhibited a greater likelihood of adhering to the interventions compared to the severely ill group. Despite incorporating various algorithms, it was not possible to predict adherence at the individual level. These findings add to the understanding of the factors that influence adherence to exercise interventions. They underscore the predictive importance of daily life functioning while indicating a lack of association between symptom severity and adherence. Future research should focus on developing targeted strategies to improve adherence, particularly for people with schizophrenia who suffer from impairments in daily functioning.

*Clinical trials registration* The study of this manuscript which the manuscript is based was registered in the International Clinical Trials Database, ClinicalTrials.gov (NCT number: NCT03466112, https://clinicaltrials.gov/ct2/show/NCT03466112?term=NCT03466112&draw=2&rank=1) and in the German Clinical Trials Register (DRKS-ID: DRKS00009804.

**Supplementary Information:**

The online version contains supplementary material available at 10.1007/s00406-024-01789-w.

## Introduction

Given the remarkable reduction in life expectancy of 10–20 years in people with schizophrenia [1–3], understanding the underlying factors contributing to their increased risk of premature death has become a critical area of research. Individuals with schizophrenia exhibit an elevated likelihood to develop diabetes [4], metabolic syndrome [5, 6], and cardiovascular diseases [3, 7]. While medication use [8] and genetic factors [9] contribute to this increased risk, lifestyle habits, including poor diet, smoking, and low physical activity levels, also exert a substantial influence [10, 11]. Therefore, addressing these factors and developing effective interventions is crucial for improving the overall health outcomes and reducing premature mortality among individuals with schizophrenia.

Integrating exercise interventions into the lives of individuals with schizophrenia holds great potential for positive outcomes. A scientometric analysis underscoring the importance of physical activity revealed a substantial body of evidence that has systematically shaped a significant research trend regarding the advantages of engaging in physical activity for preventing and treating severe mental disorders [12]. Exercise interventions reveal beneficial effects on overall cognitive performance [13–16], positive and negative symptoms [14, 17–22], depressive symptoms [14], levels of functioning [14, 19, 20], and quality of life [14, 17–19] among people with schizophrenia. Moreover, exercise interventions lead to improvements in several physical domains, including cardiovascular fitness [20, 23, 24], reduction in BMI [18, 19], and a tendency to reduce triglyceride levels [18].

In brief, exercise interventions for people with schizophrenia have a wide range of beneficial effects, covering both physical and mental domains.

However, despite their proven benefits, the issue of adherence and dropout rates is a barrier in implementing and sustaining interventions in people with schizophrenia. In particular, exercise interventions for individuals with schizophrenia are characterized by high dropout rates, spanning a range of approximately 30–80% [20, 25]. Importantly, participants cannot maximize the benefit of interventions unless they maintain adherence: for instance, substantial improvements in physical fitness, psychiatric symptoms, and overall functioning have been shown to be particularly present in individuals who successfully completed more than 50% of exercise sessions [26]. Beyond being important for clinical ameliorations of the individual patient, the dropout in clinical interventions contributes to an increased risk of re-hospitalization, which in turn increases the strain on public resources [27]. Moreover, dropout from studies introduces a strong risk for biased results, as the existing evidence relies heavily on participants who have successfully completed the intervention, potentially limiting the generalizability and validity of the findings [20].

With the evidence supporting the effectiveness of exercise interventions for individuals with schizophrenia [13–24] and the recognized difficulties in maintaining adherence to such interventions [20, 25], there is a need to identify potential predictors of adherence.

In investigations centered on exercise interventions for major depressive disorder, it has been observed that greater symptom severity [28, 29] and lower global functioning and quality of life are indicative of higher probabilities of dropout [30]. In older people, adherence to exercise interventions was positively associated with both physical ability [31] and body mass index (BMI) [32]. Further investigation into predictors of dropout from exercise interventions for people diagnosed with Parkinson’s disease indicated that the higher the cognitive functioning, the less likely was the dropout [33]. In sum, these studies highlight predictors of adherence in exercise interventions across diverse populations, including symptom severity, medication dosage, global functioning, quality of life, physical ability, BMI, and cognition.

The current study aims to enhance the understanding of potential predictors of adherence to exercise interventions in people with schizophrenia, based on the comprehensive data from a large multicenter randomized controlled clinical trial [34]. First, the influence of clinical baseline characteristics on adherence is explored, hypothesizing that higher levels of functioning, lower symptom severity, improved quality of life, lower BMI, and superior physical and cognitive scores are associated with better adherence to the exercise programs. Second, we aim to identify clinical subgroups of patients that differ in adherence. Lastly, we investigate whether adherence can be predicted on the individual level based on a combination of these various clinical characteristics.

## Methods

### Study design

The current investigation is based on data from the Enhancing Schizophrenia Prevention and Recovery through Innovative Treatments (ESPRIT) C3 study [34]. The ESPIRT C3 study is a multicenter randomized controlled trial that assessed the effects of aerobic exercise on various health outcomes in people with schizophrenia. A total of 180 participants were enrolled and randomly assigned to either aerobic endurance training (AET) or flexibility, strengthening, and balance training (FSBT). Participants in the AET group cycled on bicycle ergometers at a moderate exercise intensity level, determined through a lactate threshold test conducted prior to the intervention. Participants in the FSBT group participated in a series of exercises that addressed stretching, mobility, stability, balance, and relaxation techniques. Both groups underwent supervised exercise sessions up to three times per week, with each session lasting 40–50 min. The intervention spanned 26 weeks in total. Participants had the option to cancel training sessions without facing exclusion. After the intervention phase, there was a 26-week follow-up phase. The study was conducted at five hospitals in Germany, namely Ludwig-Maximilians-Universität Munich, Zentralinstitut für Seelische Gesundheit Mannheim, Charité Berlin, Haus der Universität Dusseldorf, and Rheinisch-Westfälische Technische Hochschule Aachen University. Further details on the study protocol, and the specific criteria for inclusion and exclusion can be found in the corresponding publication [35].

### Outcome measurements

Two outcome variables were employed to evaluate adherence. The first variable was binary and indicated whether participants dropped out during the intervention phase or not (*completion of visit 6*). The second variable was continuous and represented the *number of trainings* completed by each participant.

### Baseline characteristics

Baseline characteristics of the participants were assessed prior to the intervention onset and encompassed clinical symptom ratings, functional ratings, quality of life rating, neurocognitive ratings, and physical fitness ratings. For detailed information, see Table 1.

**Table 1:** Baseline characteristics

	Abbreviation	Assessed domains
*Clinical symptom ratings*		
Positive and negative syndrome scale [36]	PANSS total	Positive, negative, and general psychopathological symptoms
		Positive symptoms
		Negative symptoms
		Depressive symptoms
Calgary depression scale for Schizophrenia [37]	PANSS positive	
	PANSS negative	
	CDSS	
*Functioning ratings*		
Functional remission of general Schizophrenia [38]	FROGS	Functioning in daily life
Global assessment of functioning scale [39]	GAF	Overall functioning, including psychiatric symptoms
		Social and occupational functioning
Social and occupational functioning assessment scale [40]	SOFAS	
*Quality of life*		
World Health Organization quality of life assessment [41]	WHOQOL	Life satisfaction
*Neurocognitive ratings*		
Rey auditory verbal learning test [42]	VLMT	Verbal declarative memory
Digit span test [43]	DST	Short-term and working memory
Brief cognitive assessment tool for Schizophrenia [44]	B-CATS	Verbal and category fluency
Digit symbol substitution test [43]	DSST	Processing speed, and memory
Pictures of facial affect recognition test [45]	PFA	Emotion recognition
Trail marking test [46]	TMT-A, -B	Visual scanning, processing speed, cognitive flexibility, and working memory updating
*Physical fitness ratings*		
Body mass index (kg/m^2^)	BMI	Weight(kg) / height(m)^2^
International physical activity questionnaire [47]	IPAQ	Physical activity in daily life

### Statistical analysis

The data analysis for the current study was conducted in Python version 3.10.11. All statistical analyses were performed with a significance threshold of *p* = 0.05. To ensure data quality, a criterion of including features with less than 20% missing values was applied. For handling the remaining missing values, the *K*-Nearest Neighbors (KNN) imputation method [48] was employed (refer to supplementary material S1 for more details).

The neurocognitive ratings were combined to create a total cognition score (refer to supplementary materials S2).

To investigate the associations between baseline characteristics, such as clinical symptoms, functioning, quality of life, cognitive performance, and physical fitness (see Table 1), multiple Logistic Regression analyses were conducted for the outcome *completion of visit 6*, and multiple linear regression analyses were performed for the outcome *number of trainings* (refer to supplementary materials S3). The analysis incorporated several covariables, including age, gender, site, CPZ, intervention group, and years of education.

Next, principal component analysis (PCA) in combination with *K*-means clustering [49] was used to identify clinical subgroups. Once the subgroups were identified, pairwise Fisher’s tests were employed to determine if there were differences in adherence, measured by the outcome *completion of visit 6*. Furthermore, pairwise Mann–Whitney *U* tests were used to explore if the subgroups differed in adherence, as measured by the outcome *number of trainings* (refer to supplementary materials S4).

Third, the aim was to predict adherence at an individual level. Therefore, Logistic Regression models and Random Forest (RF) [50] classification models were used to predict the outcome *completion of visit 6.* In addition, Ridge Regression [51] and RF [50] Regression models were employed to predict the outcome *number of trainings* (refer to supplementary materials S5).

## Results

### Study participants

The study included a group of 180 participants with schizophrenia, comprising 103 men and 77 women with an age from 18 to 65 years. This group consisted of both inpatients and outpatients. Detailed information about the characteristics of the participants is available in Table 2.

**Table 2:** Demographic characteristics (m = mean, SD = standard deviation)

Sex	Male	103	57.22%
	Female	77	42.78%
Age		m 37.38	SD 11.97
Site	Munich	105	58.33%
	Mannheim	51	28.33%
	Berlin	14	7.78%
	Düsseldorf	7	3.89%
	Aachen	3	1.67%
Group	AET	89	49.44%
	FSBT	91	50.56%
Duration of disorder (years)		m 9.57	SD 9.82
Years of education		m 14.29	SD 3.74
Body mass index (BMI in kg/m^2^)		m 28.40	SD 5.58
Chlorpromazine equivalents (CPZ in mg)		m 466.43	SD 296.01
Positive and negative syndrome scale (PANSS total)		m 50.51	SD 11.84

Of the total participants, 74 (41.11%) successfully completed visit 6, while 106 (58.89%) did not. Furthermore, 16 participants (8.89%) were randomized, but did not undertake any training sessions. Then 73 (40.56%) subjects completed between 1 and 15 training sessions, 31 (17.22%) completed between 16 and 30 training sessions, 34 (18.89%) completed between 31 and 45 training sessions, 19 (10.56%) completed between 46 and 60 training sessions, and 7 (3.89%) completed between 61 and 75 training sessions (refer to supplementary materials S6).

### Association between baseline characteristics and adherence

**Fig. 1:**
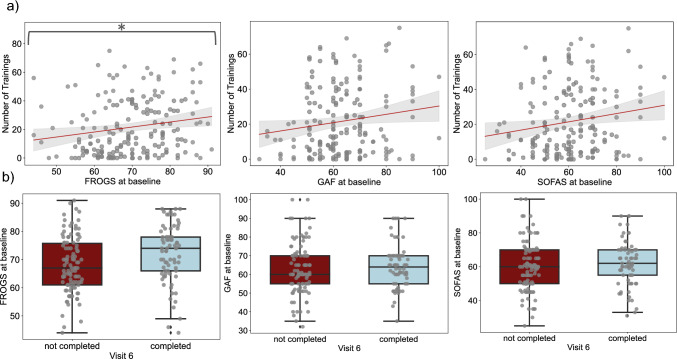
Association between functioning scores and *number of trainings* or status of visit 6. FROGS, Functional Remission of General Schizophrenia; SOFAS, Social and Occupational Functioning Assessment Scale; GAF, Global Assessment of Functioning scale. **a** These plots show the associations between baseline assessments of FROGS, SOFAS, or GAF on the x-axis and the *number of trainings* completed on the *y*-axis. Each dot in these plots represents an individual participant, the straight line represents the linear regression line fitted to the data, and the shaded area indicates the confidence interval. **b** These plots show the associations between FROGS, SOFAS, or GAF on the *x*-axis and the *number of trainings* completed on the *y*-axis. Each dot in these plots represents an individual participant

Investigations into the potential impact of baseline characteristics on the outcome variable number of training sessions revealed a significant association with the level of functioning. Among the three functioning scores analyzed, the FROGS score exhibited a statistically significant association (*β* = 0.436, CI = [0.145, 0.728], *p* = 0.004, pFDR = 0.029) with the number of training sessions. Participants with a ten-point higher score on the FROGS scale attended approximately four more training sessions on average. In addition, the SOFAS score showed a discernible trend suggesting a potential association with the number of training sessions, although it did not reach statistical significance after FDR correction (*β* = 0.246, CI = [0.043, 0.448], *p* = 0.034, pFDR = 0.070). The visualized results of the linear regression, depicting the relationship between the outcome variable *number of trainings* and the independent variables FROGS, SOFAS, and GAF, can be found in Fig. 1a.

However, other baseline characteristics, including cognition score, fitness ratings, and symptom severity, did not exhibit any significant association with the outcome variable (refer to Supplementary Material S7).

Furthermore, when investigating the association between baseline characteristics and the likelihood to complete visit 6, only the FROGS score (*β* = 0.470, CI = [0.132, 0.808], *p* = 0.006, pFDR = 0.052, OR = 1600) showed a trend after FDR correction. On average, each one-unit increase in the FROGS score was linked to a 1.6-fold increase in the odds of completing visit 6. However, the remaining functioning ratings, cognition score, fitness rating, and symptom severity ratings did not demonstrate any significant association with the *completion of visit 6* (refer to Supplementary Material S7). The visualized results of the Logistic Regression with the outcome variable *completion of visit 6* and the independent variables FROGS, SOFAS, and GAF can also be found in Fig. 1b.

### Adherence disparities among clinical clusters

To explore potential clinical patterns that might contribute to increased adherence, unsupervised clustering to identify clinical subgroups of participants was conducted.

**Fig. 2:**
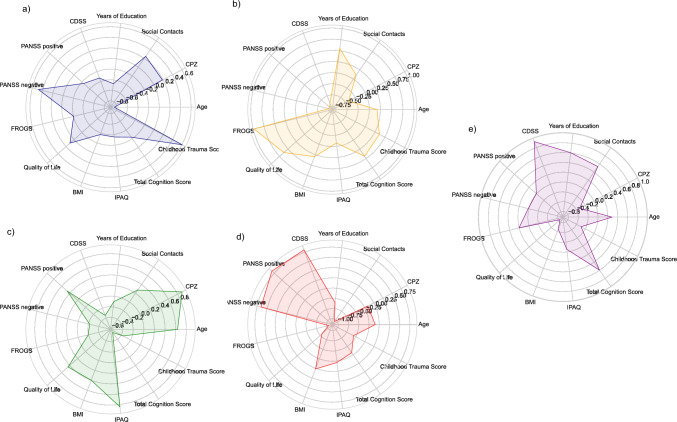
Complex radar chart of the clusters, CPZ, chlorpromazine equivalents; IPAQ, International Physical Activity Questionnaire; BMI, body mass index; FROGS, Functional Remission of General Schizophrenia; PANSS, Positive and Negative Syndrome Scale; CDSS, Calgary Depression Scale for Schizophrenia. **a** Radar chart of subgroup with pronounced negative symptoms and pronounced Childhood Trauma Score. **b** Radar chart of high-functioning and low-symptom severity subgroup. **c** Radar chart of subgroup with pronounced positive symptoms, older participants, high CPZ, and high IPAQ. **d** Radar chart of subgroup with high symptom severity and low functioning. **e** Radar chart of subgroup with pronounced depressive symptoms and low quality of life

Five distinct clusters were identified, each characterized by unique participant profiles. The initial cluster was characterized by a pattern primarily marked by negative symptoms, younger participants, and a higher Childhood Trauma Score (CTS) in comparison to the remaining clusters. In the second cluster, participants displayed elevated functional levels, lower symptom severity, and the highest quality of life compared to the other groups. The third cluster comprised older participants, who received the highest medication dosage, exhibited a higher BMI, and engaged in more physical activity than other groups. Within the fourth cluster, participants demonstrated overall higher symptom severity, lower functioning, and fewer social contacts than other clusters. The fifth cluster encompassed participants with increased depressive symptoms, coupled with a high level of cognitive performance. Figure 2 visualizes these clusters. For more details, refer to the Supplementary Material S8.

**Fig. 3:**
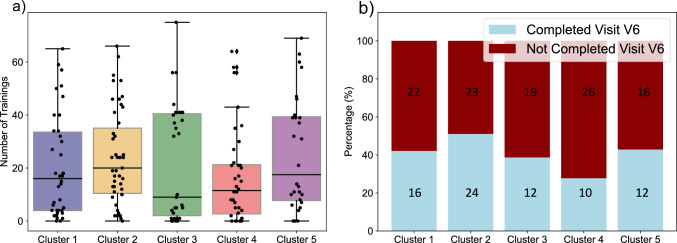
**a** Each cluster is represented by a boxplot indicating the number of completed trainings. Dots on the plot represent individual participants within the respective cluster. **b** Bar plots are provided for each cluster, illustrating the percentage of participants who completed visit V6 (lower section) and those who did not complete visit 6 (upper section). The absolute number of participants is also displayed within the bars

We identified a trend indicating that participants of cluster 2 attend more trainings than participants of cluster 4 (*p* = 0.025, pFDR = 0.249, effect size (Cohen’s d) = 0.289). In addition, there was a trend showing that participants in cluster 2 were more likely to complete visit 6 than participants in cluster 4 (*p* = 0.043, pFDR = 0.432, OR = 2.713, effect size (Cramer’s V) = 0.052) (Fig. 3).

### Individual prediction of adherence

The results of all trained models are shown in Table 3. Neither of the trained machine learning models could predict accurately, indicating that predictions at the individual level were challenging given the sample size of 180 patients. Further details on the ML analysis are provided in S9.

**Table 3:** Results of the supervised machine learning models

Model	Performance score	Train split	Test split	Standard deviation of the test split
Ridge regression	MAE	14.964	17.052	1.105
	MSE	17.962	20.535	1.270
	MRSE	4.238	4.531	0.139
	R2	0.137	− 0.144	0.194
RF regressor	MAE	11.366	16.618	0.820
	MSE	13.661	19.700	0.940
	MRSE	3.685	4.437	0.106
	R2	0.491	− 0.048	0.073
Logistic regression	Accuracy	0.674	0.554	0.076
	Balanced accuracy	0.646	0.522	0.076
	Sensitivity	0.486	0.334	0.103
	Specificity	0.806	0.710	0.081
	Precision	0.635	0.442	0.162
	F1-score	0.550	0.376	0.122
	Brier score	0.326	0.446	0.076
RF classifier	Accuracy	0.888	0.559	0.049
	Balanced accuracy	0.873	0.522	0.051
	Sensitivity	0.788	0.314	0.096
	Specificity	0.959	0.730	0.077
	Precision	0.930	0.439	0.099
	F1-score	0.851	0.360	0.086
	Brier score	0.112	0.441	0.049

## Discussion

The present study investigated the potential of clinical baseline characteristics as predictors of adherence to exercise interventions in individuals with schizophrenia. Our findings revealed that participants with higher levels of daily life functioning at baseline demonstrated better adherence, whereas symptom severity, cognitive performance, quality of life, and physical conditions did not play an important role. Analysis of clinical subgroups revealed that participants characterized by high-functioning and low-symptom severity demonstrated better adherence compared to another subgroup, which comprised individuals with low functioning and high symptom severity.

Our results suggest that mainly levels of functioning in daily life are crucial regarding adherence to exercise interventions in people with schizophrenia. A previous meta-analysis [29] investigated clinical predictors such as age, gender, disorder duration, and symptom severity, but could not find any significant associations with dropout. The current study confirms this finding and additionally identifies levels of functioning to be essential regarding adherence to exercise. Functioning directly relates to an individual’s ability to carry out daily activities and engage in social, occupational, and personal roles successfully. When a person’s functioning is compromised, they may encounter challenges in planning and executing, managing their time efficiently. For example, people with low functioning could have problems to plan their exercise schedule and to organize their way to the gym. In contrast, patients with higher symptom severity but moderate impairments in functioning may still have the capacity and social support to participate in exercise interventions.

The link between functioning and adherence to exercise interventions in individuals with schizophrenia underscores the need to support those patients with lower functioning levels in maintaining their commitment. Such support could involve various behavioral interventions, like reminders through text messages or regular telephone calls. These interventions have shown significant improvements in medication adherence [52]. Another approach to consider is a token economy system with points or financial incentives. Prior research demonstrated the effectiveness of offering financial incentives in enhancing adherence to antipsychotic depot medication among individuals diagnosed with psychotic disorders [53]. Based on our practical experience, it is advisable to establish specific, measurable, and attainable individual objectives. Special attention to goal setting and alignment for individuals with schizophrenia and lower functional levels could increase adherence to exercise programs.

In addition to the examination of single baseline characteristics such as functioning, we identified five clinical clusters of patients with schizophrenia in our sample. These clusters included a resilient functioning group, a severe symptom group, a negative symptom burden group, a depressive symptom burden group, and an active and positive symptom burden group. A previous study identified three clinical subgroups of the participating individuals with schizophrenia; a group with high negative symptoms, a distress subgroup characterized by depressive symptoms and anxiety, along with elevated positive symptoms, and a subgroup with low symptoms and high functioning [54]. And a further study, which detected psychosis subgroups, identified five subgroups termed affective psychosis, suicidal psychosis, depressive psychosis, high-functioning psychosis, and severe psychosis [55]. The subgroups identified in the current work share several similarities with the subgroups found in these studies. In both, the present study and the earlier research, clinical subgroups based on the severity of specific symptoms, such as negative symptoms, depressive symptoms, and positive symptoms, were obtained. In addition, the concept of high-functioning subgroups is evident in both the current study and the earlier research.

When investigating which subgroup demonstrated better adherence to the exercise intervention, a notable trend emerged, indicating that the high-functioning group exhibited higher levels of exercise engagement and were more likely to complete the intervention compared to the severely ill group. These findings supported the idea that the level of functioning plays a crucial role in adherence to exercise interventions. As outlined above, the benefits of higher functioning, such as enhanced planning abilities and adherence to training appointments, can lead to the observed association. Surprisingly, the high-functioning and low-symptom group did not exhibit a distinct advantage in adherence compared to the groups with pronounced negative symptoms and pronounced CTS or pronounced positive symptoms. In these three subgroups, the level of functioning was very similar. The finding suggests that if the level of functioning is sufficiently high and exclusively negative, positive, or depressive symptoms are present, it did not seem to hinder adherence to the exercise intervention.

Attempts to utilize supervised machine learning models for generating individual predictions based on a combination of baseline characteristics resulted in suboptimal outcomes. The performance of these models in terms of classification was only marginally better than chance. Moreover, the results of the regression analysis indicated that the models’ performance was inferior to a simple prediction based on the mean of the outcome variable. These findings suggest overfitting, wherein the models perform well on the training dataset but poorly on the test dataset.

This phenomenon indicates a limitation of the current study. The limited size of the dataset is a challenge when applying machine learning techniques robustly [56]. The potential consequences of overfitting are reflected in poor generalization to the test data, ultimately contributing to the unsatisfactory results observed in the study. Despite having a relatively large dataset with a considerable number of participants, it is important to acknowledge that its size was not sufficient to run complex machine learning algorithms. A larger dataset would be necessary to ensure more reliable results and increase the generalizability of the findings. Furthermore, it is noteworthy that other potential predictors could influence adherence to exercise interventions. These include not only the intensity and duration of the intervention, motivation, and the expertise of the professionals administering the exercise program [29], but also factors like satisfaction with the training, preferences for specific exercises, and the perceived subjective benefits of the intervention. Another potential determinant influencing adherence to exercise interventions may be the patient’s status as either an inpatient or outpatient, as indicated by a recent meta-analysis highlighting the stronger effects of exercise interventions in outpatients compared to inpatients [57]. Interestingly, in our sample, symptom severity did not play a significant role in determining adherence. Therefore, it can be assumed that the distinction between inpatient and outpatient status may not be a crucial factor affecting adherence. A further limitation of the present study is the impact of the COVID-19 pandemic, as some participants may have been unable to attend training sessions due to infection or related limitations. This external factor introduces a potential bias in the adherence and completion rates observed in the study.

In conclusion, the present study revealed a positive association between higher levels of functioning and adherence to exercise interventions among individuals with schizophrenia. Enhancing adherence to exercise interventions is crucial, as these interventions offer multiple benefits in schizophrenia. Future research should focus on strategies to improve adherence, particularly for individuals with schizophrenia who have lower levels of functioning. Possible approaches may involve sending session reminders and considering the implementation of a token economy. Exploring and implementing such strategies may help to improve adherence rates and maximize the effectiveness of exercise interventions for individuals with schizophrenia.

### Supplementary Information

Below is the link to the electronic supplementary material.

Supplementary file1 (DOCX 3364 KB)

## Data Availability

All analysis scripts and documentation sheets can be made available upon request.
