# Supplementary material for: Predictors of adherence to exercise interventions in people with schizophrenia
Source: Eur Arch Psychiatry Clin Neurosci. 2024 Mar 29;274(6):1265–76. doi: 10.1007/s00406-024-01789-w (PMC11362191; doi:10.1007/s00406-024-01789-w)
Supplement: Supplementary file 1 — Supplementary file1 (DOCX 3364 KB) [file 406_2024_1789_MOESM1_ESM.docx]

**Supplementary Materials**

**Predictors of Adherence to Exercise Interventions in People with Schizophrenia**

Rebecca Schwaiger^1,12^, Isabel Maurus^1,12^, Moritz Lembeck^1,12^, Irina Papazova^2,12^, David Greska^1,12^, Susanne Muenz^1,12^, Eliska Sykorova^3,13^, Cristina E. Thieme^3,13^, Bob O. Vogel^4,13^, Sebastian Mohnke^4,13,14^, Charlotte Huppertz^6^, Astrid Roeh^2,12^, Katriona Keller-Varady^7^, Berend Malchow^8^, Henrik Walter^4,13^, Bernd Wolfarth^5,13^, Wolfgang Wölwer^9^, Karsten Henkel^6^, Dusan Hirjak^3,13^, Andrea Schmitt^1,10,12^, Alkomiet Hasan^2,12^, Andreas Meyer-Lindenberg^3,13^, Peter Falkai^1,11,12^ & Lukas Roell^1,12^*

^1^Department of Psychiatry and Psychotherapy, LMU University Hospital, LMU Munich, Munich, Germany

^2^Department of Psychiatry, Psychotherapy and Psychosomatics of the University Augsburg, Medical Faculty, University of Augsburg, Bezirkskrankenhaus Augsburg, Augsburg, Germany-­­

^3^Central Institute of Mental Health, Medical Faculty Mannheim, Heidelberg University, Germany

^4^Department of Psychiatry and Psychotherapy, University Hospital Charité Berlin, Berlin, Germany

^5^Department of Sports Medicine, University Hospital Charité Berlin, Berlin, Germany

^6^Department of Psychiatry, Psychotherapy and Psychosomatics, RWTH Aachen University, Germany

^7^Hannover Medical School, Clinic of Rehabilitation and Sports Medicine, Hannover, Germany

^8^Department of Psychiatry and Psychotherapy, University Hospital Göttingen, Göttingen, Germany

^9^Department of Psychiatry and Psychotherapy, Medical Faculty, Heinrich-Heine University, Düsseldorf, Germany

^10^Laboratory of Neuroscience (LIM27), Institute of Psychiatry, University of Sao Paulo, São Paulo, Brazil

^11^Max Planck Institute of Psychiatry, Munich, Germany

^12^DZPG (German Center for Mental Health) Munich/Augsburg

^13^DZPG (German Center for Mental Health)

^14^ Psychotherapy Practice, Pichelsdorfer Str. 73, 13595 Berlin

***Corresponding author:**

Lukas Roell, M.Sc.

Department of Psychiatry and Psychotherapy

University Hospital, LMU Munich,

Nussbaumstrasse 7, 80336 Munich, Germany

Tel: +49-(0)89-4400-55537

Fax: +49-(0)89-4400-55530

E-mail: Lukas.Roell@med.uni-muenchen.de

**S1 Missing values**

Features with less than 20% missing values were included in the analysis. Consequently, the feature *aerobic fitness range*, which was determined by the achieved wattage on the ergometer at a lactate concentration between 1.8-2.5 mmol/L divided by body weight, was excluded due to its high percentage of missing values (42.78%). A detailed overview of all the included features and their corresponding missing values can be found in Table 1.

**Table 1:** Missing values of the used features

| **Feature** | **Number of missing values** | **Percentage of missing values** |
| --- | --- | --- |
| **Demographic characteristics**  Gender  Age  Site  Group  Duration of disorder  Years of education  CPZ | 0  0  0  0  2  2  2 | 0  0  0  0  1.11  1.11  1.11 |
| **Clinical symptom ratings**  PANSS total  PANSS negative  PANSS positive  CDSS | 0  0  0  0 | 0  0  0  0 |
| **Functioning ratings**  FROGS  GAF  SOFAS | 5  0  0 | 2.78  0  0 |
| **Quality of life**  WHOQOL | 17 | 9.44 |
| **Neurocognitive ratings**  Total cognition score | 24 | 13.33 |
| **Physical fitness ratings**  BMI  IPAQ | 1  12 | 0.56  6.67 |

To address missing values, the K-Nearest Neighbors (KNN) imputation method was used and was implemented using the scikit-learn package [1]. KNN offers several strengths, such as being a non-parametric approach that does not rely on assumptions about the underlying data distribution. It leverages local information to capture patterns within the dataset. In determining the number of neighbors (k), the square root of the number of complete cases [2] was utilized. In this case, since there were 122 complete cases, we chose eleven as the number of neighbors.

In predicting individual-level adherence, the KNN imputation method was applied within a nested cross-validation framework to address information leakage concerns.

**S2 Calculation of neurocognitive ratings**

The included tests for the neurocognitive ratings are listed in Table 2.

**Table 2:** Neurocognitive ratings with their abbreviation and subtests

| Test | Abbreviation | Subtests |
| --- | --- | --- |
| Brief Cognitive Assessment Tool for Schizophrenia [3] | B-CATS | B-CATS Animals  B-CATS Fruits  B-CATS Vegetables |
| Rey Auditory Verbal Learning Test [4] | VLMT | VLMT int.  VLMT1  VLMT6  VLMT7  VLMT diff. |
| Trail Marking Test [5] | TMT | TMT-A  TMT-B |
| Digit Span Test [6] | DST | DST forwards  DST backwards |
| Pictures of Facial Affect Recognition Test [7] | PFA | - |
| Digit Symbol Substitution Test[6] | DSST | - |

The total cognition score was determined through the following steps. Firstly, missing values were imputed using the k-Nearest Neighbors method [6] to address any gaps in the data. Next, to maintain consistency with other neurocognitive ratings, the VLMT diff., TMT A, and TMT B scores were multiplied by -1. This adjustment was necessary as these measures have scales that are opposite to the other neurocognitive ratings. To ensure comparability, all ratings were standardized using z-scores.

Subsequently, the test scores were computed by calculating the mean value of the respective subtests. For instance, the BCATS score was obtained by averaging the scores from its three subtests: BCATS animals, BCATS vegetables, and BCATS fruits. This approach was also applied to the VLMT, TMT, and DST, along with their respective subtests.

Finally, the total cognition score was derived by taking the mean of the calculated BCATS, VLMT, TMT, DST, PFA, and DSST scores. Hence, the formula for the total cognition score can be expressed as follows:

$$cognition total=\bar{\bar{BCATS}+\bar{VLMT}+\bar{TMT}+\bar{DST}+DSST+PFA}$$

**S3 Details of the statistical analysis of the associations between clinical baseline characteristics and adherence**

A comprehensive assessment was conducted to evaluate the impact of each baseline characteristic on adherence. Multiple logistic regressions were performed, with the outcome variable *completion of visit 6* serving as the dependent variable and each baseline characteristic acting as an independent variable. The baseline characteristics included PANSS total, PANSS negative, PANSS positive, CDSS, FROGS, GAF, SOFAS, WHOQOL, total cognitive score, BMI, and IPAQ. Additionally, control variables such as age, gender, site, dose of chlorpromazine equivalents (CPZ), intervention group, and education years were included in the analysis. The dataset was preprocessed to handle missing values through KNN-imputation [2]. To ensure comparability and eliminate the impact of scale differences, the data was z-standardized before performing the calculations. Furthermore, multiple linear regressions were performed, with the outcome variable *number of training* sessions as the dependent variable, and each baseline characteristic as the independent variable. The same set of control variables was included in these analyses. However, the dataset was not z-standardized for these calculations. The p-values of the regression models for the three PANSS scores, the three functioning ratings, and the two physical fitness ratings were corrected using the Benjamini-Hochberg method [8] to address the issue of multiple comparisons and control the false discovery rate.

**Section 4: Statistical Analysis of Subgroups**

The primary aim of this approach was to investigate adherence patterns and identify potential variations within subsets of participants using unsupervised machine learning methods. The analysis incorporated various baseline characteristics, including PANSS positive, PANSS negative, CDSS, FROGS, WHOQOL, BMI, IPAQ, total cognition score, age, CPZ, social contacts, education years, and CTS. We chose to use FROGS as the functioning assessment score because it is more precise than GAF due to its more specific criteria. Additionally, we selected PANSS positive and PANSS negative over PANSS total as they provide more informative insights into the clusters when compared to using PANSS total alone. To ensure data integrity, the site effect was addressed through the application of the combat technique [9] using the pyComat package [10]. Subsequently, KNN-imputation and z-standardization were employed with the Standard Scaler from scikit-learn preprocessing package [1] for handling missing values and ensuring comparability across variables, respectively (see S1).

The preprocessed data underwent Principal Component Analysis (PCA) using the scikit-learn package [1]. The identification of key components was based on the Kaiser's rule [11], defining components with eigenvalues above one (refer to Fig. 1). In our analysis, five components had eigenvalues above one, providing further insights into the data. The top five features of each component were visualized (see Fig. 2) to enhance understanding.

Subsequently, the identified key components were used for clustering using the K-Means algorithm [12]. K-Means is a centroid-based approach that assigns data points to clusters based on distance from centroids on the dimension-reduced data [13, 14]. For the K-Means algorithm [12], we set various parameters, including n_clusters to define the number of clusters, init (k-means++) for intelligent centroid initialization, n_init (auto) for multiple runs with different centroid seeds, and random_state (=111) for reproducibility of centroid initialization.

To determine the appropriate number of clusters, we employed the Davies-Bouldin Score [15], using the scikit-learn package in Python [1] (see Fig. 3). The Davies-Bouldin Score evaluates clustering quality by measuring the similarity between clusters based on the ratio of within-cluster distances to between-cluster distances. Lower values indicate better clustering [15]. Multiple cluster configurations, ranging from two to seven clusters, were investigated to find the optimal solution. The analysis revealed that the configuration with five clusters yielded the lowest score, suggesting the best clustering solution. We further conducted a thorough examination of resulting clusters to ensure their plausibility, analyzing the characteristics and patterns exhibited within each cluster to ensure meaningful groupings.

Once the clustering was completed, we assessed whether the clusters exhibited differences in the two outcome variables. To analyze differences regarding the outcome variable *number of trainings*, a pairwise Mann-Whitney U test [16] was employed. For the analysis of differences regarding the outcome variable *completion of visit 6,* a pairwise Fisher's test was conducted [17]. The p-values obtained from these tests were adjusted for multiple comparisons using the Benjamini-Hochberg method [8].


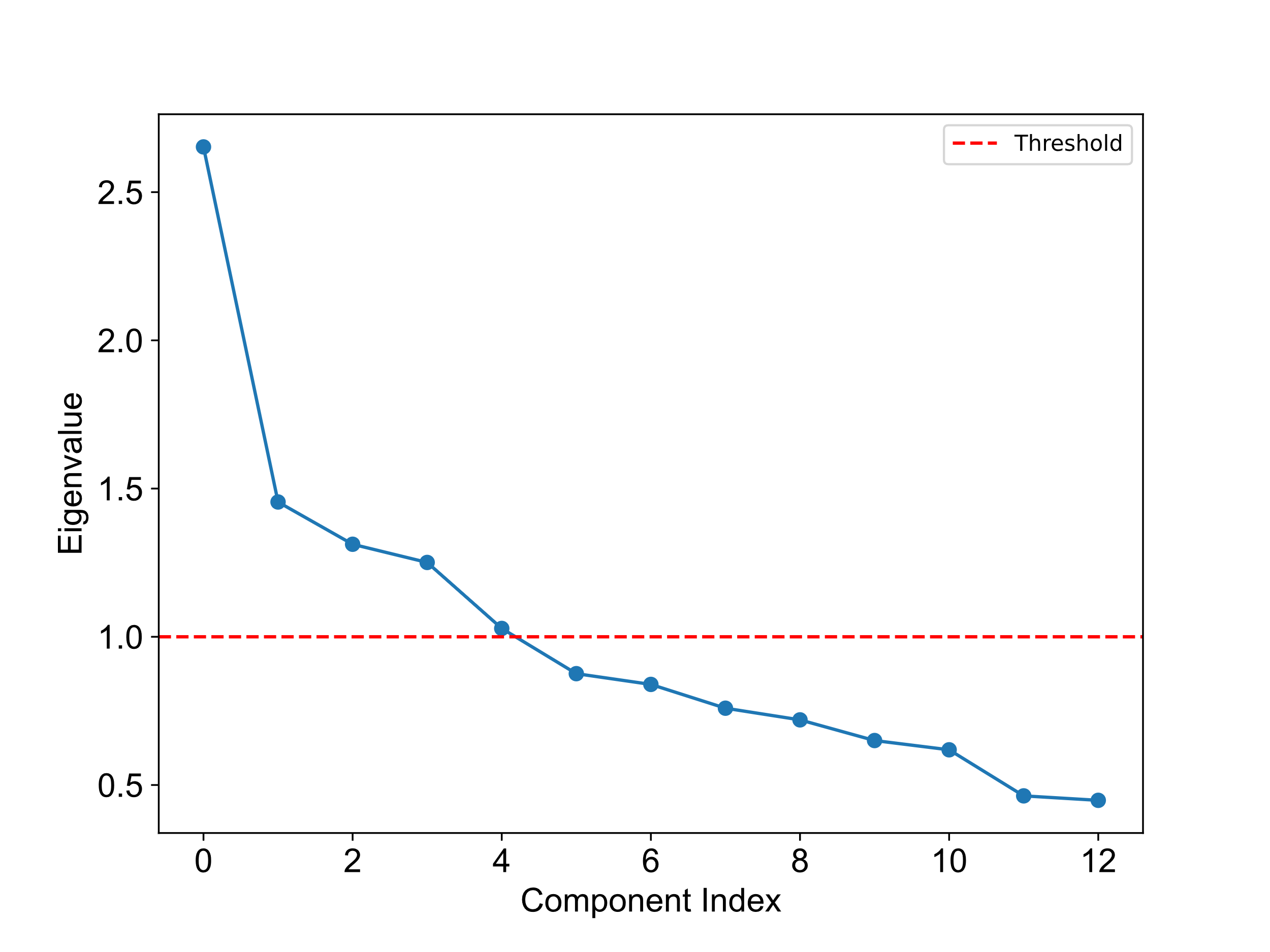


**Fig. 1** Eigenvalues of the 13 principal components

This figure presents a connected line graph illustrating the eigenvalues of the 13 principal components. Each dot on the graph represents an eigenvalue, with the x-axis denoting the component index and the y-axis representing the magnitude of the eigenvalues. The dashed horizontal line indicates the threshold of one, as defined by Kaiser's rule.


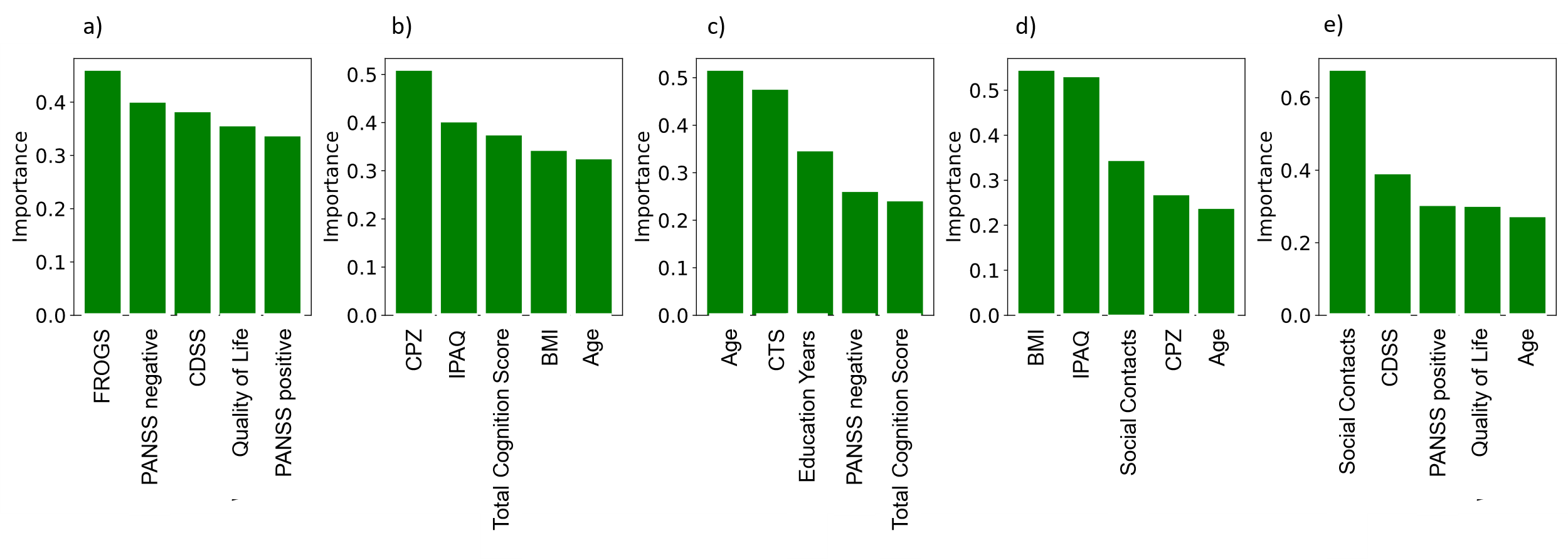


**Fig. 2** Top five features of each principal component of the PCA. FROGS, Functional Remission of General Schizophrenia; PANSS, Positive and Negative Syndrome Scale; CDSS, Calgary Depression Scale for Schizophrenia; CPZ, Chlorpromazine equivalents; IPAQ, International Physical Activity Questionnaire; BMI, body mass index; CTS, childhood trauma score. a) First component of the PCA with its five most important features. b) Second component of the PCA with its five most important features. c) Third component of the PCA with its five most important features. d) Fourth component of the PCA with its five most important features. e) Fifth component of the PCA with its five most important features.


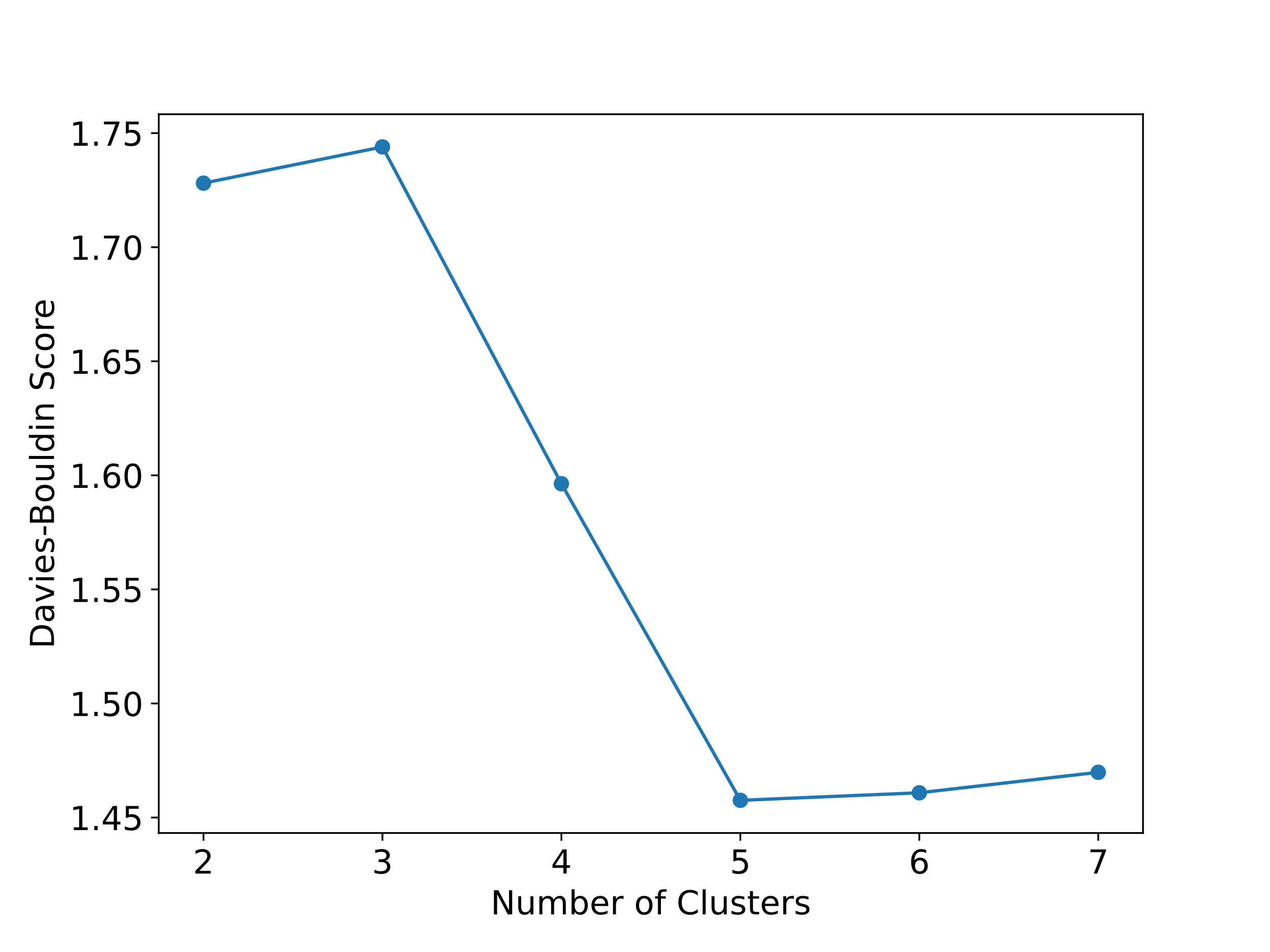


**Fig. 3** Davies-Bouldin score for different cluster configurations.

**S5 Details of the** **statistical analysis of the prediction of adherence on individual level**

The aim of this analysis was to assess the predictive capability of a combined set of seventeen baseline characteristics on individual adherence. The factors included age, CPZ, social contacts, years of education, CDSS, PANSS positive, PANSS negative, FROGS, quality of life, BMI, IPAQ, total cognition score, CTS, group, gender, relatives with mental illness, and site. To ensure comparability, the following preprocessing steps were applied: KNN-imputation for handling missing values (see S1), z-standardization for continuous variables, and encoding of categorical variables using numerical dummy variables for inclusion in the machine learning models.

For the investigation scikit-learn [1] were utilized, a package for implementing supervised machine learning algorithms. For the continuous outcome of the *number of trainings*, Ridge Regression [18] was employed, a linear regression technique with L2 regularization to prevent overfitting [19]. For the binary outcome *completion of visit 6*, Logistic Regression with L2 regularization [20] was used. Additionally, Random Forest (RF) Regressor and RF Classifier models were employed for predicting the number of trainings and determining the completion of visit 6, respectively. RF is a machine learning method that combines multiple decision trees to create a robust model [21]. Several attempts were made with the RF models:

1. Without hyperparameter tuning and feature selection.
2. With hyperparameter tuning using grid search optimization [22].
3. With feature selection using Recursive Feature Elimination (RFE) [23]
4. With both hyperparameter tuning and feature selection.

RFE is a technique that systematically removes less important features from a model based on the feature importance derived from the RF. In this case, ten features were selected using RFE [23]. To optimize the Random Forest model, Grid Search CV [24] was utilized for hyperparameter tuning. Hyperparameters considered were n_estimators (number of trees in the forest), max_depth (maximum depth of each tree), max_features (number of features to evaluate for the best split), min_samples_split (minimum number of samples required to split an internal node), and min_samples_leaf (minimum number of samples needed to form a leaf node). Two values for each hyperparameter were chosen to minimize computational resources. Additionally, the random_state parameter was used to control the randomness of the model.

Nested cross-validation [25] as employed for all models, with an inner loop of five iterations and an outer loop of ten iterations, repeated ten times. The inner loop handled hyperparameter selection, while the outer loop provided an unbiased estimate of the chosen evaluation score using the selected hyperparameters. Nested cross-validation helps mitigate overfitting, maximizes data utilization, and provides a more reliable estimate of the model's performance, particularly beneficial for smaller datasets [25, 26].

For interpretation purposes, a range of evaluation scores was used. For classification, the accuracy score measured the overall correctness of predictions made by the classifier with the test data [27]. Since the dataset was imbalanced, the balanced accuracy score was also used, providing a more balanced evaluation for imbalanced classes [28]. Additionally, sensitivity, specificity, precision, and F1-Score [27] were evaluated. The Brier score, considering calibration and statistical consistency between predicted probability and observation, was also used for evaluation [29, 30]. For regression, the following scores were used: the mean absolute error (MAE) [31], mean squared error (MSE) [32], the mean root squared error (MRSE) [32], and R-squared (R2) [33], which represents the proportion of the variance in the dependent variable explained by the independent variable.

**S6 Measurements of adherence to exercise interventions**


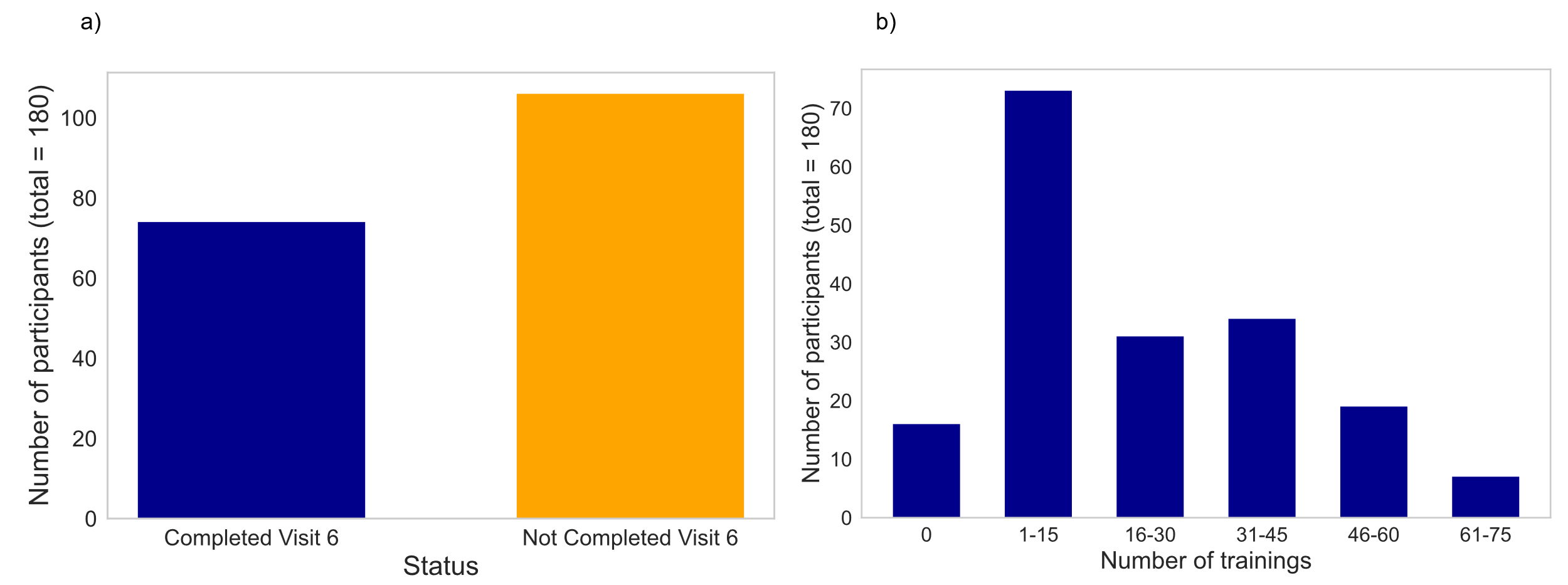


**Fig. 4** Measurements of Adherence to Exercise Interventions. a) Visit 6 Completion Status. b) Distribution of Completed Trainings

**S7 Association between baseline characteristics and adherence**

**Table 3:** Association between baseline characteristics and adherence

| **Outcome** | **Baseline characteristic** | **β** | **CI low** | **CI high** | **p-value** | **pFDR** | **Odds Ratio** |
| --- | --- | --- | --- | --- | --- | --- | --- |
| **Number of trainings** | **Clinical symptom ratings**  PANSS total  PANSS negative  PANSS positive  CDSS | -0.182  -0.529  -0.087  -0.561 | -0.434  -1.150  -0.852  -1.280 | 0.070  0.093  0.677  0.158 | 0.156  0.095  0.822  0.126 | 0.426  0.253  0.924  - | -  -  -  - |
|  | **Functioning ratings**  FROGS  GAF  SOFAS | 0.436  0.246  0.246 | 0.145  0.018  0.043 | 0.728  0.474  0.448 | ****0.004**  ***0.034**  ***0.018** | ***0.029**  0.137  0.070 | -  -  - |
|  | **Quality of life**  WHOQOL | 1.43 | -2.215 | 5.078 | 0.439 | - | - |
|  | **Neurocognitive ratings**  Cognition score | 1.907 | -4.529 | 8.342 | 0.559 | - | - |
|  | **Physical ratings**  BMI  IPAQ | 0.289  0.000 | -0.238  -0.001 | 0.817  0.001 | 0.280  0.536 | 0.647  0.715 | -  - |
| **Completion of visit 6** | **Clinical symptom ratings**  PANSS total  PANSS negative  PANSS positive  CDSS | -0.254  -0.298  -0.118  -0.188 | -0.581  -0.635  -0.432  -0.516 | 0.073  0.039  0.195  0.140 | 0.127  0.083  0.459  0.261 | 0.510  0.333  0.728  - | 0.775  0.742  0.889  0.829 |
|  | **Functioning ratings**  FROGS  GAF  SOFAS | 0.470  0.077  0.133 | 0.132  -0.228  -0.174 | 0.808  0.383  0.429 | ****0.006**  0.620  0.397 | 0.052  0.771  0.749 | 1.600  1.080  1.142 |
|  | **Quality of life**  WHOQOL | 0.146 | -0.167 | 0.459 | 0.361 | - | 1.157 |
|  | **Neurocognitive ratings**  Cognition score | 0.065 | -0.268 | 0.397 | 0.704 | - | 1.067 |
|  | **Physical ratings**  BMI  IPAQ | 0.306  -0.325 | -0.011  -0.720 | 0.624  1.938 | 0.058  0.076 | 0.234  0.303 | 1.358  0.683 |

**S8 Details of the baseline characteristics of each subgroup**


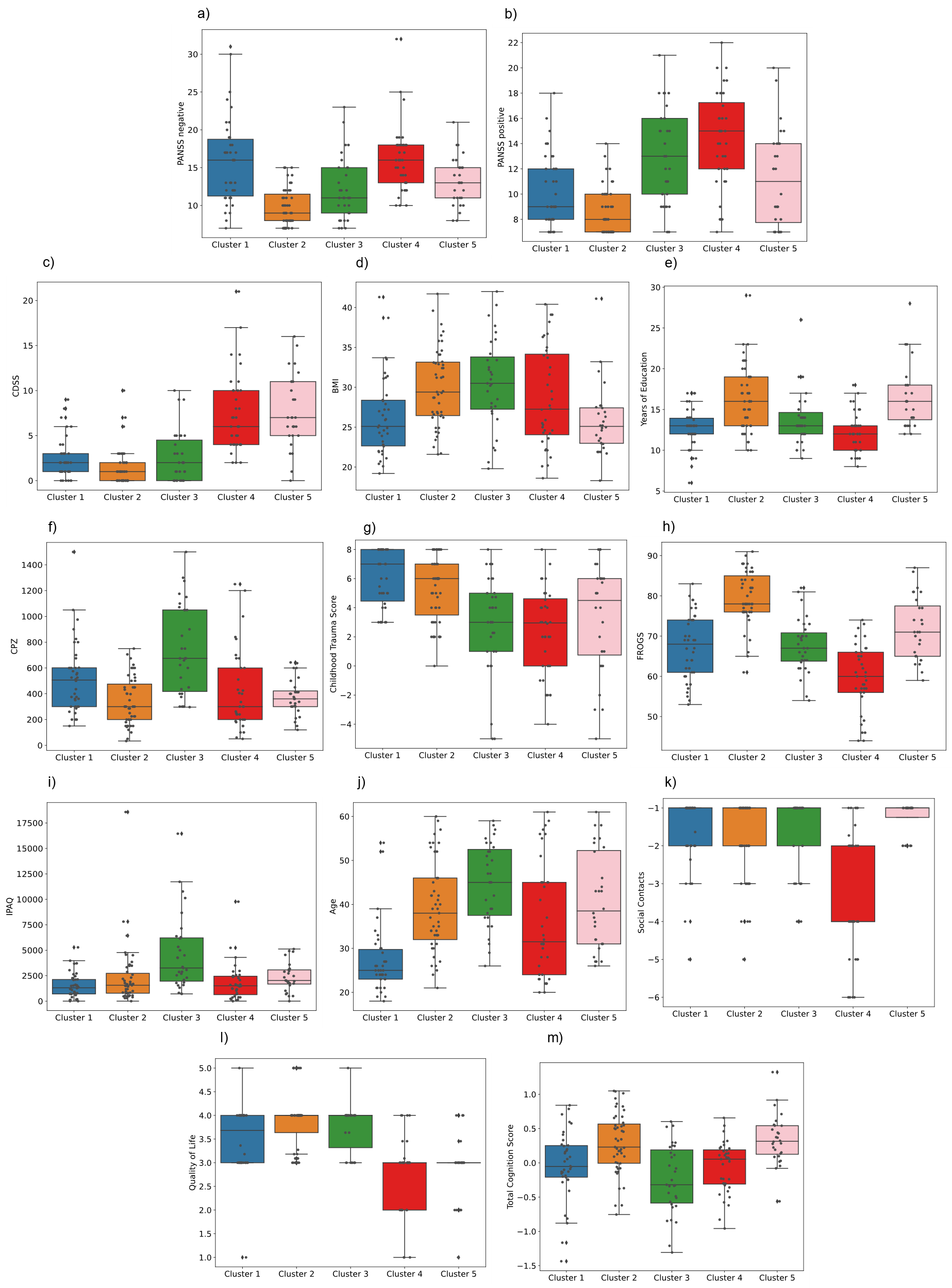


**Fig. 5** Boxplots of Baseline Characteristics within Each Subgroup. PANSS, Positive and Negative Syndrome Scale; CDSS, Calgary Depression Scale for Schizophrenia; BMI, body mass index; CPZ, Chlorpromazine equivalents; FROGS, Functional Remission of General Schizophrenia; IPAQ, International Physical Activity Questionnaire.

The figures show the distribution of:

a) PANSS negative scores within the clusters.

b) PANSS positive scores within the clusters.

c) CDSS scores within the clusters.

d) BMI values within the clusters.

e) years of education within the clusters.

f) CPZ values within the clusters.

g) CTS scores within the clusters.

h) FROGS scores within the clusters.

i) IPAQ values within the clusters.

j) age within the clusters.

k) social contacts within the clusters.

l) quality of life within the clusters.

m) total cognition score within the clusters.

Each boxplot provides insights into the distribution of the corresponding baseline characteristic for each subgroup, facilitating comparisons and identifying potential variations among the clusters.

**S9: Random Forest Models**

Our approach was to investigate if prediction via supervised machine learning models is possible on individual level. We investigated multiple RF models, with or without feature selection and hyperparameter tuning. The results of the models in *Table 4* shows that the Random Forest models overfitted. The overfitting was stronger for the models without features selection and without hyperparameter tuning, see *Table 4.*

**Table 4:** Further results of the supervised machine learning models

|  |  | **Mean results of the inner loop** | **Mean results of the outer loop** |
| --- | --- | --- | --- |
| **RF Regressor** | - without feature selection - without hyperparameter tuning | MAE: 6.245  MSE: 7.568  MRSE: 2.751  R2: 0.847 | MAE: 17.165  MSE: 20.322  MRSE: 4.506  R2: -0.116 |
|  | - with feature selection - without hyperparameter tuning | MAE: 6.114  MSE: 7.506  MRSE: 2.739  R2: 0.849 | MAE: 16.712  MSE: 20.078  MRSE: 4.479  R2: -0.090 |
|  | - without feature selection - with hyperparameter tuning | MAE: 12.895  MSE: 15.346  MRSE: 3.917  R2: 0.369 | MAE: 16.508  MSE: 19.571  MRSE: 4.422  R2: -0.034 |
| **RF Classifier** | - without feature selection - without hyperparameter tuning | accuracy: 1.000  balanced accuracy: 1.000  sensitivity: 1.000  specificity: 1.000  precision: 1.000  F1 score: 1.000  Brier score: 0.000 | accuracy: 0.562  balanced accuracy: 0.521  sensitivity: 0.286  specificity: 0.755  precision: 0.449  F1 score: 0.346  Brier score: 0.438 |
|  | - with feature selection - without hyperparameter tuning | accuracy: 1.000  balanced accuracy: 1.000  sensitivity: 1.000  specificity: 1.000  precision: 1.000  F1 score: 1.000  Brier score: 0.000 | accuracy: 0.552  balanced accuracy: 0.513  sensitivity: 0.291  specificity: 0.735  precision: 0.433  F1 score: 0.344  Brier score: 0.448 |
|  | - without feature selection - with hyperparameter tuning | accuracy: 0.901  balanced accuracy: 0.887  sensitivity: 0.807  specificity: 0.967  precision: 0.944  F1 score: 0.868  Brier score: 0.099 | accuracy: 0.576  balanced accuracy: 0.534  sensitivity: 0.295  specificity: 0.773  precision: 0.479  F1 score: 0.357  Brier score: 0.424 |

RF uses a feature importance metric to assess the importance of each feature. For classification, importance is measured using the Gini impurity, which evaluates the contribution of each feature to node splitting. Features with higher importance have a greater impact on classification accuracy. For regression, feature importance in RF is determined by measuring the mean decrease in impurity. It quantifies the strength to which each feature reduces the variance produced by node splitting. Features with higher importance play a more important role in reducing the variance within the predicted continuous variable. [34]

Fig. 6 illustrates the feature importance of the RF models. Notably, the scores for symptom severity emerge as the most important features, indicating their significant influence on the model's predictions. Additionally, the FROGS score exhibits relative importance, ranking as the third most important feature for the Regression model and the fourth most important feature for the Classifier model. However, exercising caution in interpreting the feature importance is crucial, given the suboptimal performance of the model.


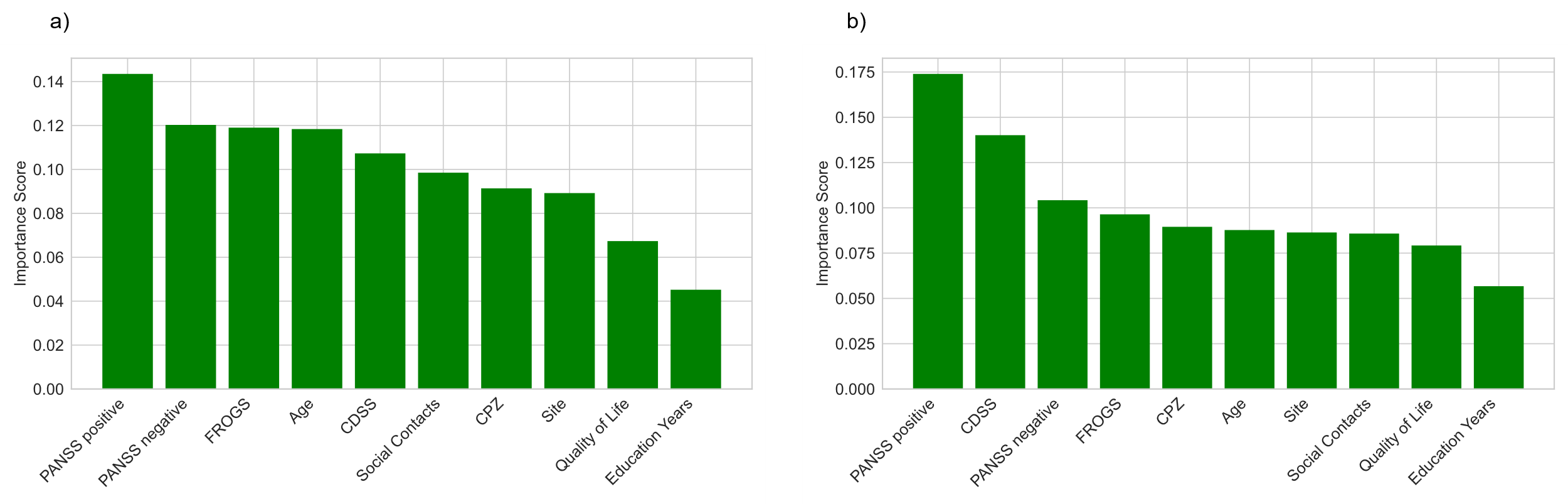


**Fig. 6** Feature Importance of the RF Models. PANSS, Positive and Negative Syndrome Scale; FROGS, Functional Remission of General Schizophrenia; CDSS, Calgary Depression Scale for Schizophrenia; CPZ, Chlorpromazine equivalents. a) This figure illustrates the feature importance of the RF Regression model. b) This figure shows the feature importance of the RF Classifier model.

References

1. Fabian Pedregosa, Gaël Varoquaux, Alexandre Gramfort, Vincent Michel, Bertrand Thirion, Olivier Grisel, Mathieu Blondel, Peter Prettenhofer, Ron Weiss, Vincent Dubourg, Jake Vanderplas, Alexandre Passos, David Cournapeau, Matthieu Brucher, Matthieu Perrot, Édouard Duchesnay Scikit-learn: Machine Learning in Python. Journal of Machine Learning Research 2011(12):2825–2830

2. Jonsson P, Wohlin C (2004) An evaluation of k-nearest neighbour imputation using likert data:108–118. https://doi.org/10.1109/METRIC.2004.1357895

3. Hurford IM, Marder SR, Keefe RSE, Reise SP, Bilder RM (2011) A brief cognitive assessment tool for schizophrenia: construction of a tool for clinicians. Schizophrenia bulletin 37(3):538–545. https://doi.org/10.1093/schbul/sbp095

4. Vakil E, Blachstein H (1993) Rey auditory-verbal learning test: Structure analysis. J. Clin. Psychol. 49(6):883–890. https://doi.org/10.1002/1097-4679(199311)49:6<883:AID-JCLP2270490616>3.0.CO;2-6

5. Reed JC, Reed HBC The Halstead—Reitan Neuropsychological Battery:93–129. https://doi.org/10.1007/978-1-4757-9820-3_4

6. Tewes U (1991) HAWIE-R. Hamburg-Wechsler-Intelligenztest für Erwachsene, Revision 1991 ; Handbuch und Testanweisung, 1. Aufl. Huber-Psychologie-Tests. Huber, Bern, Stuttgart, Toronto

7. Ekman P, Friesen WV (1974) Detecting deception from the body or face. Journal of Personality and Social Psychology 29(3):288–298. https://doi.org/10.1037/h0036006

8. Benjamini Y, Hochberg Y (1995) Controlling the False Discovery Rate: A Practical and Powerful Approach to Multiple Testing. Journal of the Royal Statistical Society. Series B (Methodological) 57(1):289–300. https://doi.org/10.1111/j.2517-6161.1995.tb02031.x

9. Johnson WE, Li C, Rabinovic A (2007) Adjusting batch effects in microarray expression data using empirical Bayes methods. Biostatistics (Oxford, England) 8(1):118–127. https://doi.org/10.1093/biostatistics/kxj037

10. Behdenna A, Haziza J, Azencott C-A, Nordor A (2020) pyComBat, a Python tool for batch effects correction in high-throughput molecular data using empirical Bayes methods. https://doi.org/10.1101/2020.03.17.995431

11. Kaiser HF (1960) The Application of Electronic Computers to Factor Analysis. Educational and Psychological Measurement 20(1):141–151. https://doi.org/10.1177/001316446002000116

12. Hartigan JA, Wong MA (1979) Algorithm AS 136: A k-means clustering algorithm. Journal of the royal statistical society. series c (applied statistics) 28(1):100–108

13. Ding C, He X (2004) K -means clustering via principal component analysis:29. https://doi.org/10.1145/1015330.1015408

14. Mohr PE, Cheng CM, Claxton K, Conley RR, Feldman JJ, Hargreaves WA, Lehman AF, Lenert LA, Mahmoud R, Marder SR, Neumann PJ (2004) The heterogeneity of schizophrenia in disease states. Schizophrenia research 71(1):83–95. https://doi.org/10.1016/j.schres.2003.11.008

15. Davies DL, Bouldin DW (1979) A Cluster Separation Measure. IEEE Trans. Pattern Anal. Mach. Intell. PAMI-1(2):224–227. https://doi.org/10.1109/TPAMI.1979.4766909

16. Nachar N (2008) The Mann-Whitney U: A Test for Assessing Whether Two Independent Samples Come from the Same Distribution. TQMP 4(1):13–20. https://doi.org/10.20982/tqmp.04.1.p013

17. Upton GJG (1992) Fisher's Exact Test. Journal of the Royal Statistical Society. Series A (Statistics in Society) 155(3):395. https://doi.org/10.2307/2982890

18. Hoerl AE, Kennard RW (1970) Ridge Regression: Applications to Nonorthogonal Problems. Technometrics 12(1):69–82. https://doi.org/10.1080/00401706.1970.10488635

19. Ying X (2019) An Overview of Overfitting and its Solutions. J. Phys.: Conf. Ser. 1168:22022. https://doi.org/10.1088/1742-6596/1168/2/022022

20. Ng AY Feature selection, L1 vs. L2 regularization, and rotational invariance:78. https://doi.org/10.1145/1015330.1015435

21. Breiman L (2001) Random Forests. Machine Learning 45(1):5–32. https://doi.org/10.1023/A:1010933404324

22. Wang X, Gong G, Li N, Qiu S (2019) Detection Analysis of Epileptic EEG Using a Novel Random Forest Model Combined With Grid Search Optimization. Frontiers in human neuroscience 13:52. https://doi.org/10.3389/fnhum.2019.00052

23. Díaz-Uriarte R, Alvarez de Andrés S (2006) Gene selection and classification of microarray data using random forest. BMC bioinformatics 7:3. https://doi.org/10.1186/1471-2105-7-3

24. Shekar BH, Dagnew G Grid Search-Based Hyperparameter Tuning and Classification of Microarray Cancer Data:1–8. https://doi.org/10.1109/ICACCP.2019.8882943

25. Stone M (1974) Cross-Validatory Choice and Assessment of Statistical Predictions. Journal of the Royal Statistical Society. Series B (Methodological) 36(2):111–147

26. Wainer J, Cawley G (2021) Nested cross-validation when selecting classifiers is overzealous for most practical applications. Expert Systems with Applications 182:115222. https://doi.org/10.1016/j.eswa.2021.115222

27. M H, M.N S (2015) A Review on Evaluation Metrics for Data Classification Evaluations. IJDKP 5(2):1–11. https://doi.org/10.5121/ijdkp.2015.5201

28. Brodersen KH, Ong CS, Stephan KE, Buhmann JM The Balanced Accuracy and Its Posterior Distribution:3121–3124. https://doi.org/10.1109/ICPR.2010.764

29. Rufibach K (2010) Use of Brier score to assess binary predictions. Journal of clinical epidemiology 63(8):938-9; author reply 939. https://doi.org/10.1016/j.jclinepi.2009.11.009

30. Brier GW (1950) Verification of forecasts expressed in terms of probability. Monthly weather review 78(1):1–3

31. Armstrong JS Evaluating Forecasting Methods 30:443–472. https://doi.org/10.1007/978-0-306-47630-3_20

32. Makridakis S, Andersen A, Carbone R, Fildes R, Hibon M, Lewandowski R, Newton J, Parzen E, Winkler R (1982) The accuracy of extrapolation (time series) methods: Results of a forecasting competition. J. Forecast. 1(2):111–153. https://doi.org/10.1002/for.3980010202

33. Maddala GS (1983) Limited-dependent and qualitative variables in econometrics, vol 3. Cambridge university press

34. Grömping U (2009) Variable Importance Assessment in Regression: Linear Regression versus Random Forest. The American Statistician 63(4):308–319. https://doi.org/10.1198/tast.2009.08199
